# Supplementary material for: Glycan-mediated enhancement of reovirus receptor binding
Source: Nat Commun. 2019 Oct 1;10:4460. doi: 10.1038/s41467-019-12411-2 (PMC6773860; doi:10.1038/s41467-019-12411-2)
Supplement: Supplementary file 1 — Supplementary Information [file 41467_2019_12411_MOESM1_ESM.pdf]

Supplementary Information for

Glycan-mediated enhancement of reovirus receptor binding

Koehler et al.

This PDF file includes:

Supplementary Table 1

Supplementary Figures 1 to 9

Other Supplementary Materials for this manuscript includes the following:

Supplementary Movies 1 to 4

Supplementary Table 1| Statistical analysis of the number of bonds established between JAM-A cell-surface receptors and T3SA+ virions under different conditions. P values were derived from comparisons of data before and after injection of the sialylated glycan (Neu5Ac, LSTa) or non-sialylated glycan (LNnT). The number of bonds established after injection of sialylated glycans differs significantly in comparison with the data before or after injection of the non-sialylated glycan. ns, $P > 0.05$; **, $P < 0.01$; ***, $P < 0.001$; ****, $P < 0.0001$; determined by two-sample t-test in Origin. For all experiments, data are representative of at least n=15 cells from n=5 independent experiments.

# bonds	+ Neu5Ac	+ LSTa	+ LNnT
**I**	ns	ns	ns
**II**	****	****	ns
**III**	**	**	ns
**IV**	****	***	ns
**V**	****	****	ns
**VI**	****	****	ns

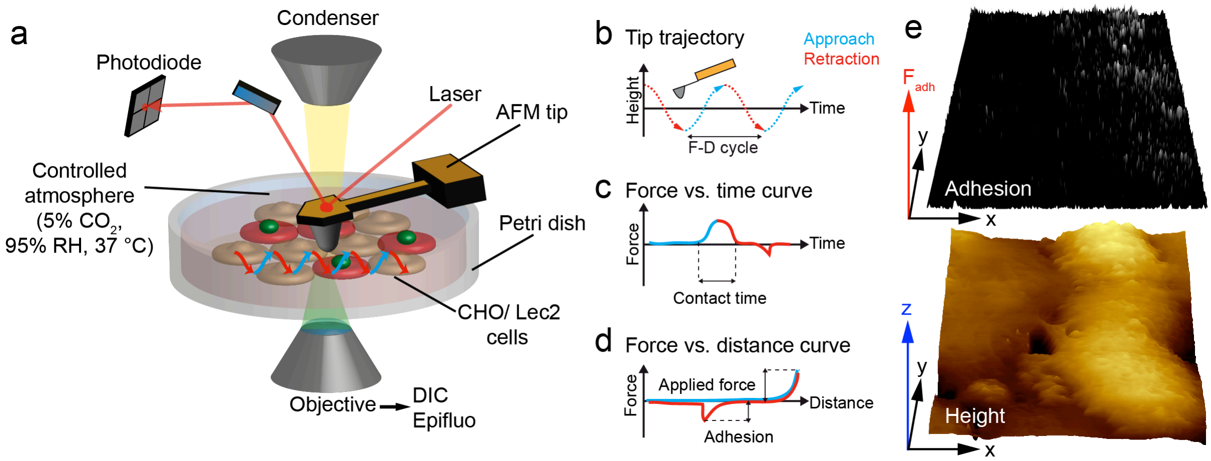

Supplementary Figure 1| Principle of FD-based AFM to probe reovirus binding to living cells. (**a**) The AFM is placed on an optical microscope. CHO or Lec2 cells are maintained in a specially designed cell culture chamber, which allows control of temperature and the gas atmosphere and prevents the medium from evaporation. (**b**) The AFM cantilever, bearing the tip functionalized with the virus of interest, is oscillated with frequency in the kHz range with a sinusoidal driving motion inducing approach and retraction movements towards the sample. (**c, d**) The recorded tip-sample interactions are displayed as force vs. time (**c**) or force vs. distance, (**d**) which allows tracking of forces established towards the biological sample. (**e**) Mechanical properties (including adhesion) can be extracted from individual force curves and directly correlated with their position on the sample (e.g., height image and corresponding adhesion map).

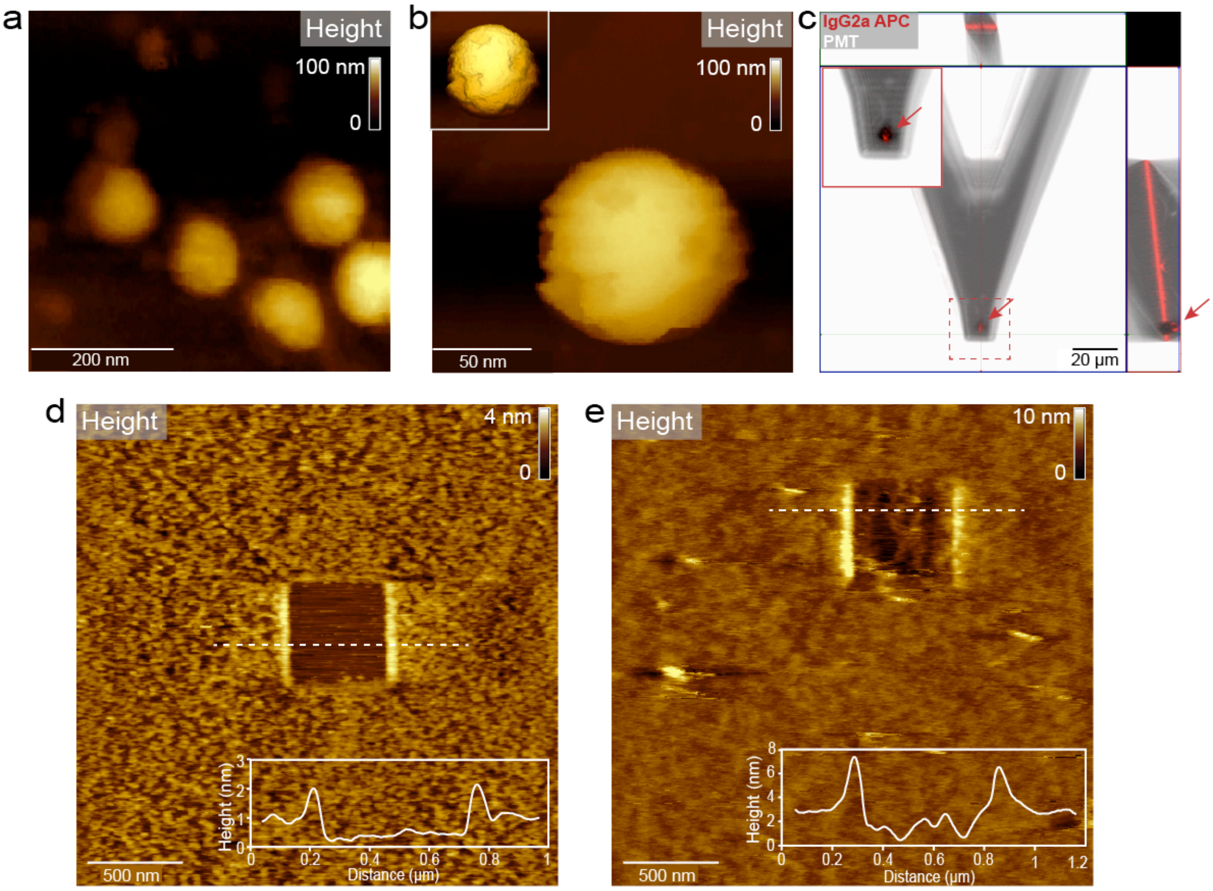

Supplementary Figure 2| Characterization of reovirus particles and validation of tip and surface immobilization. (**a, b**) AFM height images of reovirus particles deposited on freshly cleaved HOPG substrate at low (**a**) and high (**b**) magnification. Insert: 3D reconstruction. (**c**) Z-stack image of an AFM tip functionalized with reovirus obtained by laser-scanning optical microscopy after staining with primary antibody against reovirus and APC-conjugated secondary antibody (red). The inset image highlights the virion link at the tip apex. Experiments were repeated three times with similar results. (**d, e**) AFM topography image of a SA (**d**) or JAM-A (**e**) coated surface after scanning a 500 x 500 nm area at high forces (~ 18 nN) to remove the attached biomolecules (referred to as "scratching" experiment). Insets: Cross-sections taken along the white dashed line in **d** and **e**, showing an accumulation of biological materials on the sides of the square. The biomolecule-free surface of inside the square was ~ 1 nm (**d**) or ~ 2 nm (**e**) lower than the surrounding biomolecule-coated surface, providing an estimate of the thickness of SA (**d**) or JAM-A (**e**) deposited layer.

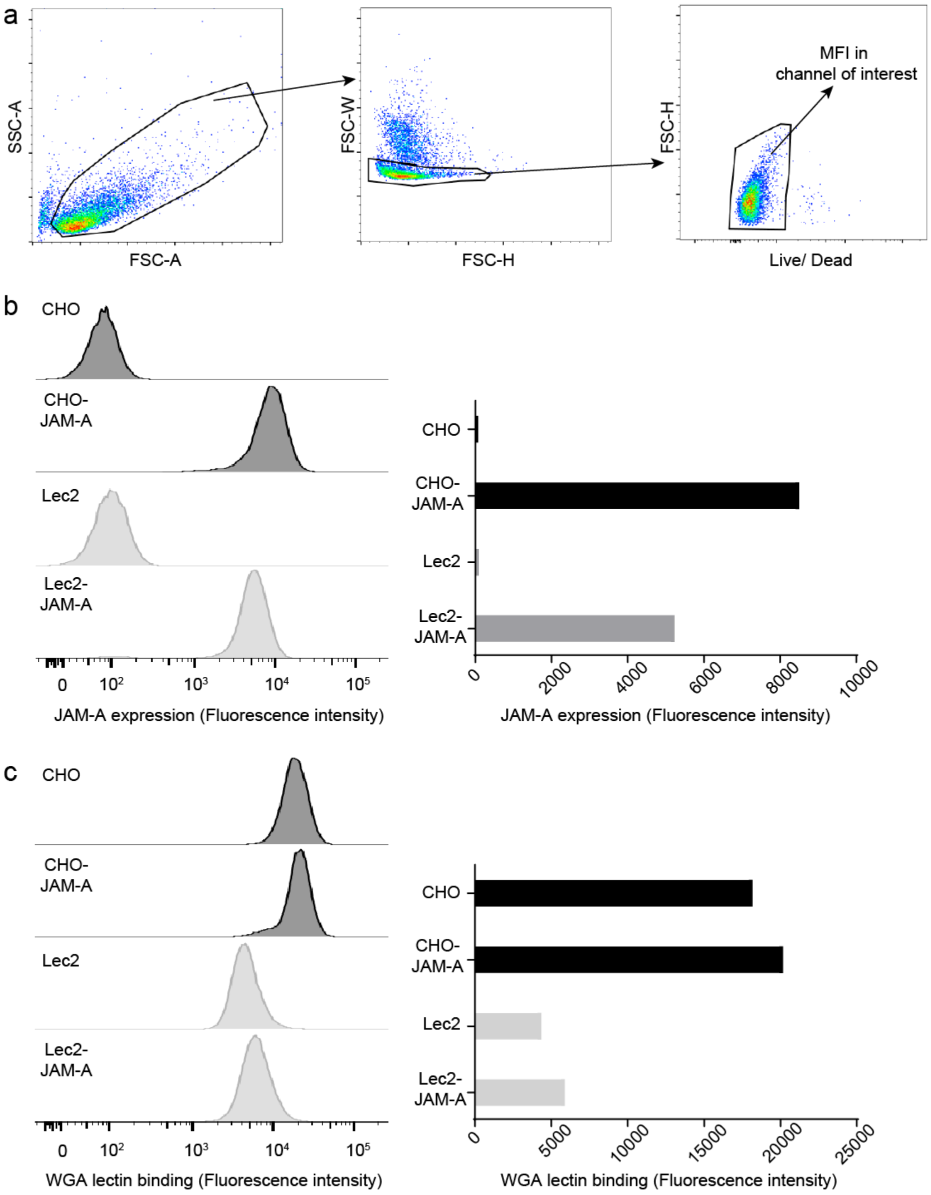

Supplementary Figure 3| Characterization of cell surface receptor expression by cell lines used in the study. (**a**) Gating strategy used for flow cytometry analysis created from a representative data set. In the first two gating steps, forward and side scatter were used to select for single cells, which were subsequently gated for live cells using the LIVE/DEAD fixable violet dead cell stain kit (Invitrogen). Median fluorescence intensity (MFI) of live cells in the channel of interest was then determined. (**b**) Flow cytometry profiles (left) and corresponding quantification of median fluorescence intensities (right) of JAM-A expression. JAM-A was detected using a monoclonal antibody and indirect immunofluorescence. (**c**) CHO and Lec2 cell lines were analyzed for expression of cell-surface sialic acid by incubation with fluorescent lectin (wheat germ agglutinin, WGA). Graphs show cytometry profiles of WGA bound to indicated cell lines (left) and quantification of median fluorescence intensity of bound lectin (right).

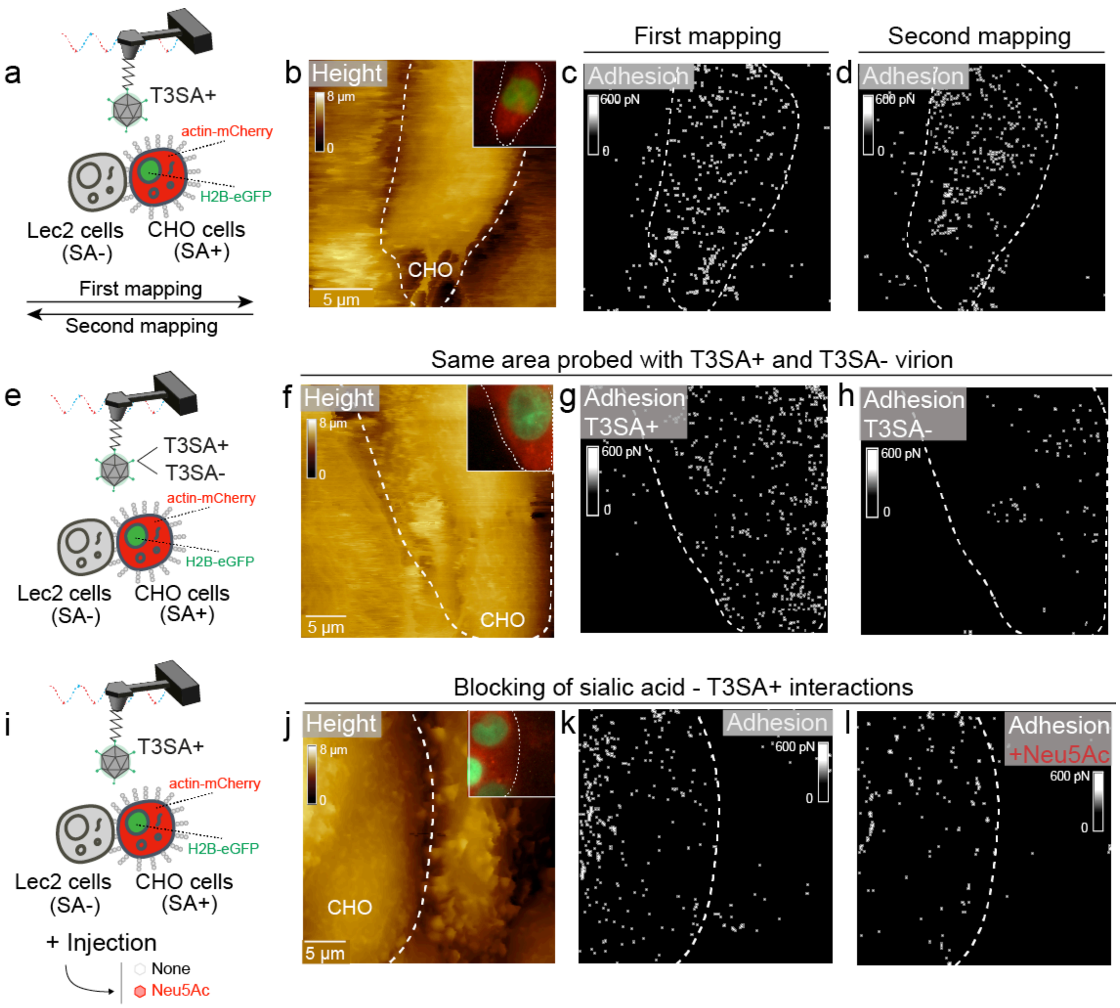

Supplementary Figure 4| Control experiments for studying the SA contribution in reovirus binding to living cells. (**a-d**) Consecutive mapping of T3SA+ virus binding to the cell-mixture show similar results. (**a**) Cartoon of the experiment highlighting that CHO cells are fluorescently labeled. FD-based AFM height image (**b**) (25 µm x 25 µm fluorescent image of the cells) and corresponding adhesion channels show similar results for two consecutive maps (**c, d**), indicating that the virus was firmly attached to the tip and did not degrade over time. (**e-h**) Same areas on the cell were consecutively probed with T3SA+ and T3SA- virions. (**e**) Cartoon of the experiment. (**f**) FD-based AFM height image and corresponding adhesion forces, acquired first with T3SA+ virions on the tip (**f, g**), followed by scanning the same area with T3SA- virions on the tip (**h**). The significant decrease in adhesion (white pixels) on CHO cells after changing the tip to the non-SA-binding virus supports the specificity of probing cell-surface sialic acid interactions with T3SA+. As another control experiment for SA-specific binding, blocking studies were conducted to test inhibition of T3SA+ interactions (as shown in **i-n**). (**i**) Cartoon of the experiment. (**j**) FD-based AFM height image and corresponding adhesion forces acquired first with T3SA+ virions on the tip (**j, k**), followed by scanning the same area after injection of 1 mM Neu5Ac (**l**) that can bind to and block reovirus interaction with cell surface SA. A significant reduction of adhesion events can be seen. All AFM images were acquired using an oscillation frequency of 0.25 kHz and amplitude of 750 nm, under cell culture conditions. Experiments were repeated 5-10 times. For higher visibility, the pixel size in the adhesion image was enlarged two-fold.

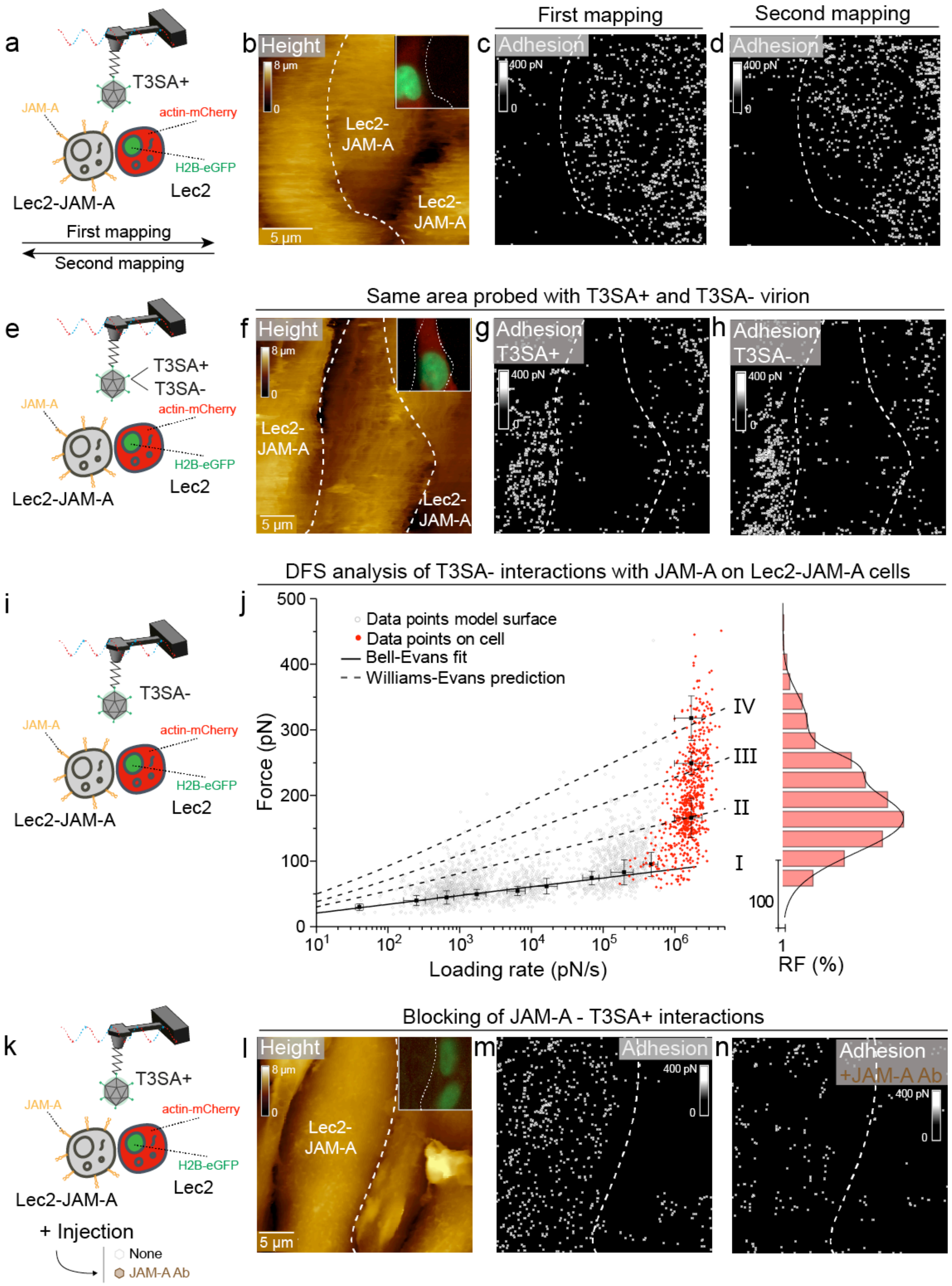

Supplementary Figure 5| Control experiments for studying the contribution of JAM-A in reovirus binding to living cells. (**a-d**) Consecutive mappings of T3SA+ virus binding to Lec2 and Lec2-JAM-A cell-mixture show similar results. (**a**) Cartoon of the experiment highlighting that Lec2 cells are fluorescently labeled. FD-based AFM height image (**b**) (25 µm x 25 µm

fluorescent image of the cells is shown in inset) and corresponding adhesion channels show similar results for two consecutive maps (**c, d**), indicating that the virus was firmly attached to the tip and did not degrade over time. (**e-h**) Same areas on cells were probed with first a T3SA+ virion and then with a T3SA- virion. (**e**) Cartoon of the experiment. (**f**) FD-based AFM height image and corresponding adhesion channels, acquired first with T3SA+ virions on the tip (**f, g**), followed by scanning the same area with T3SA- virions on the tip (**h**). Both adhesion images show similar results, indicating that JAM-A is engaged in reovirus binding independent of the presence of SA binding site on the virus. (**i-j**) DFS analysis of T3SA- interactions with JAM-A extracted from adhesion areas on Lec2-JAM-A cells. (**i**) Cartoon of the experiment. (**j**) DFS plot of T3SA- interactions with JAM-A on model surfaces (grey circles, taken from Fig. 4b – lower panel) and living cells (red dots). Histogram of the force distribution observed on cells fitted with a multi-peak Gaussian distribution (n=620 data points) is shown on the side. Error bars indicate s.d. of the mean value. (**k-n**) As another control experiment for JAM-A-specific binding, the effect of cell-surface-receptor-blocking reagents on T3SA+ interactions was tested. (**k**) Cartoon of the experiment. (**l-n**) FD-based AFM height images and corresponding adhesion images, acquired first with T3SA+ virions on the tip without blocking reagents (**l, m**), followed by scanning the same area after injection of 10 µg/ml JAM-A Ab (**n**) to block cell-surface JAM-A molecules. A significant reduction of adhesion events was observed. All AFM images were acquired using an oscillation frequency of 0.25 kHz and amplitude of 750 nm under cell culture conditions. Experiments were repeated 5-10 times. For higher visibility, the pixel size in the adhesion image was enlarged two-fold. Source data are provided as a Source Data file.

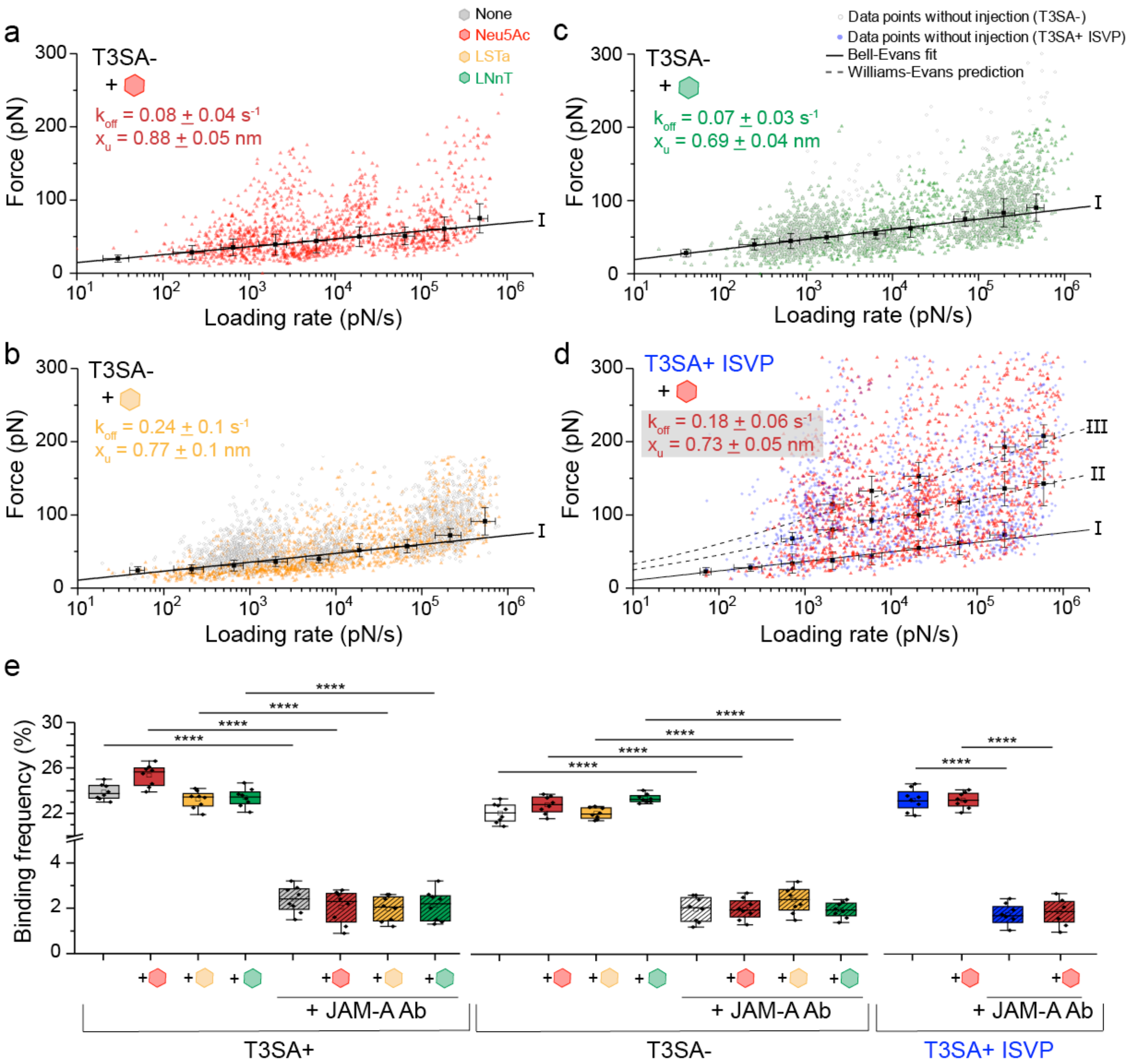

Supplementary Figure 6| Testing the effect of free SA compounds on T3SA- binding to JAM-A. (**a**-**c**) DFS plots of the interaction between T3SA- and JAM-A after adding 1 mM Neu5Ac (**a**, red), 1 mM LSTa (**b**, yellow), or 1 mM LNnT (lacking SA group) (**c**, green) probed on model surfaces. Overlaid grey circles represent the binding events before injection of the compounds. Single JAM-A-T3SA- interactions are observed in all four experiments and fitted with the Bell-Evans model (black line). In contrast to the results shown in Fig. 6, injection of Neu5Ac or LSTa does not induce any change in JAM-A-T3SA- binding or establishment of multivalent interactions, indicating that the sialic acid binding site in T3SA+ is responsible for this observation. (**d**) DFS plot of the interaction between T3SA+ ISVP and JAM-A after adding 1 mM Neu5Ac (red) probed on model surfaces. Neu5Ac does not induce any change in the multivalent binding behavior from that observed in the absence of free glycan (blue dots, also shown in Fig. **4e**). (**e**) Box plot of BF observed for JAM-A-T3SA+ (left panel), JAM-A-T3SA- (middle panel), and JAM-A-T3SA+ ISVP (right panel) interactions, without SA compounds (grey for T3SA+, white for T3SA-, and blue for T3SA+ ISVP) and after adding Neu5Ac (red), LSTa (yellow), or LNnT (green), as well as after injection of 10 µg/ml JAM-A Ab as a receptor-

blocking reagent (dashed lines in the respective boxes). The horizontal line within the box indicates the median, boundaries of the box indicate the 25th- and 75th- percentile, and the whiskers indicate the highest and lowest values of the results. The square in the box indicates the mean. The observed reduction in binding frequency in the presence of JAM-A Ab confirms the specificity of the observed interactions. For all experiments, data are representative of at least n=3 independent experiments. ****, $P < 0.0001$; determined by two-sample t-test in Origin. Error bars indicate s.d. of the mean value. Source data are provided as a Source Data file.

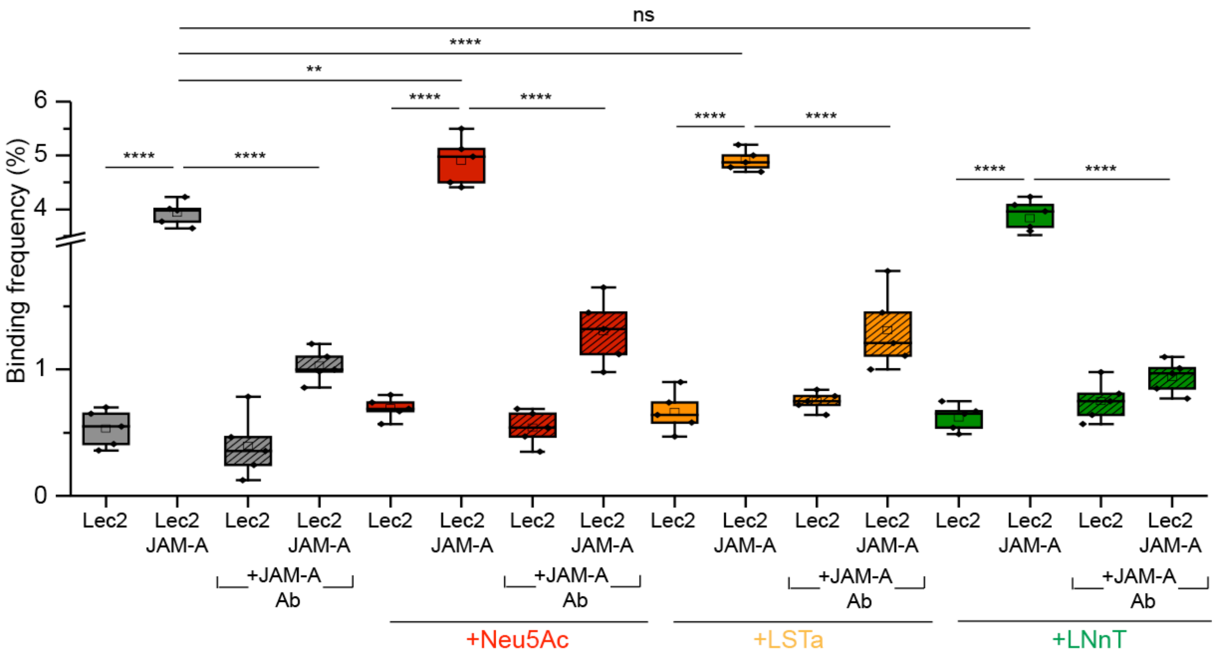

Supplementary Figure 7| Monitoring the effect of SA addition on reovirus binding to living cells. Box plot of the BF observed for T3SA+ virions with (dashed lines) and without injection of JAM-A Ab (10 µg/ml) as well as after adding the indicated glycans. The horizontal line within the box indicates the median, boundaries of the box indicate the 25th- and 75th-percentile, and the whiskers indicate the highest and lowest values of the results. The square in the box indicates the mean. Data are representative of at least n=5 independent experiments. ns, $P > 0.05$; **, $P < 0.01$; ****, $P < 0.0001$; determined by two-sample t-test in Origin. Source data are provided as a Source Data file.

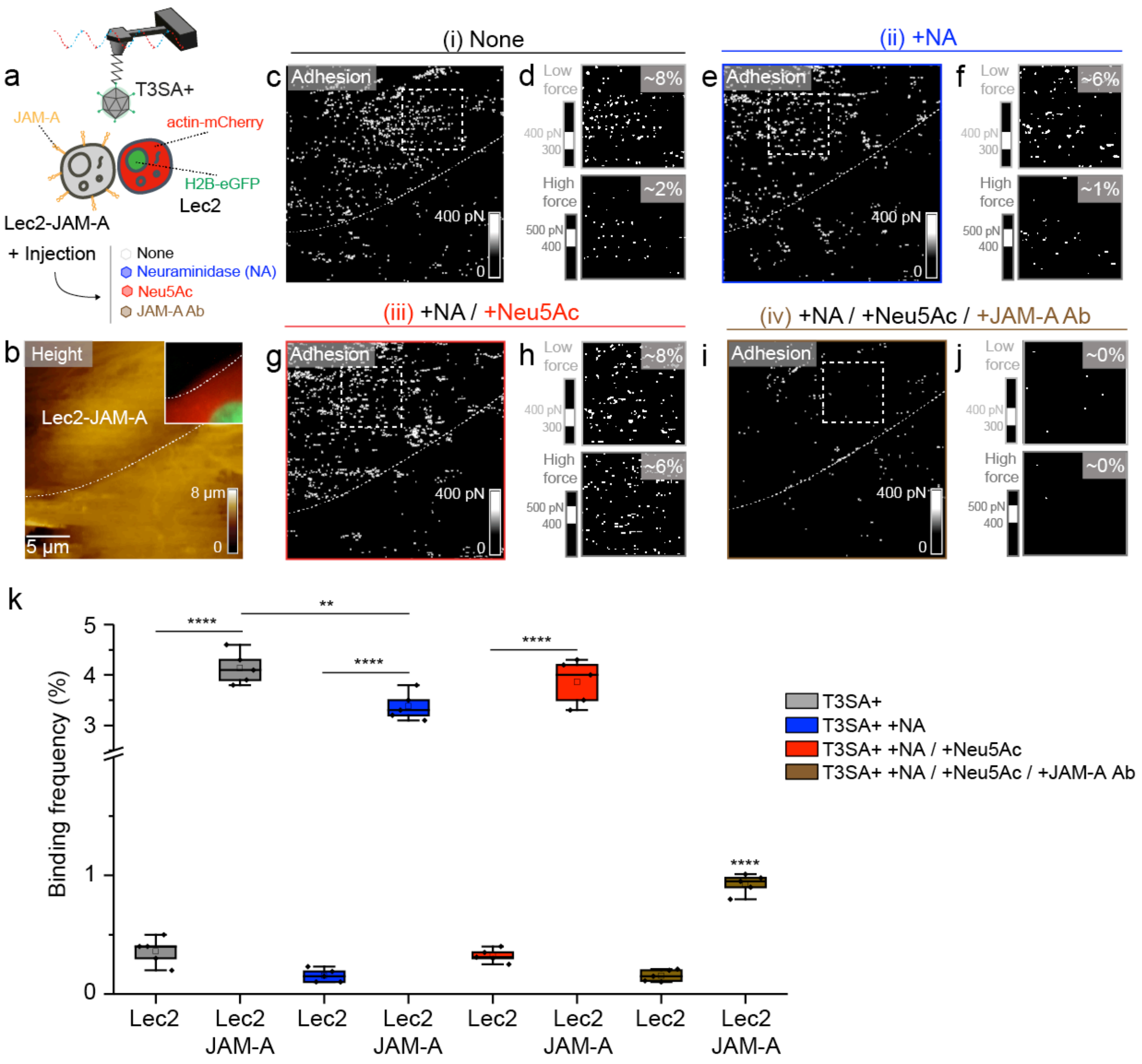

Supplementary Figure 8| Monitoring the effect of SA addition on reovirus binding to living cells after neuraminidase treatment. (**a**) Cartoon of the experiment highlights that Lec2 cells are fluorescently labeled and shows the order of the injections. (**b-j**) FD-based AFM height image (25 µm x 25 µm fluorescent image of the cells is shown in inset) (**b**) and corresponding adhesion channels, acquired first in growth medium (**c**), followed by scanning the same area after neuraminidase treatment (**e**) to remove remaining α-SA on the cell surface. A slight decrease ($P < 0.01$) in adhesion events is observed, indicating that NA treatment removed residual SA on the cell surface. (**d,f**) Enlarged images of adhesion maps recorded on Lec2-JAM-A cells (dashed square in adhesion map). The upper images display the lower force range (300 to 400 pN), whereas the lower images display the higher force range (400 to 500 pN), with significantly fewer adhesion events before and after NA treatment. The frequency of adhesion events is indicated. After NA treatment, free Neu5Ac (1 mM) was added, and the same area was rescanned (**g**). Enlarged images of adhesion maps recorded on Lec2-JAM-A cells (dashed square in adhesion map and similar areas as in **c,e** show more adhesion events in the high force range following injection of sialylated glycan. This result is concordant with the experiment conducted using cells without NA treatment (**Fig. 7a-e**). As a final step, the same area was scanned after injection of 10 µg/ml JAM-A Ab (**I, j**) to block

cell-surface JAM-A molecules. A significant reduction in adhesion events was observed. All AFM images were acquired using an oscillation frequency of 0.25 kHz and amplitude of 750 nm under cell-culture conditions. Experiments were repeated 3-5 times. For clarity and better visibility, the pixel size in the adhesion images were enlarged two-fold. (**k**) Box plot of the BF observed for T3SA+ virions first without treatment (grey), followed by NA treatment (blue), addition of free Neu5Ac (red), and finally, injection of JAM-A Ab (brown). The horizontal line within the box indicates the median, boundaries of the box indicate the 25th- and 75th- percentile, and the whiskers indicate the highest and lowest values of the results. The square in the box indicates the mean. Data are representative of at least n=4 independent experiments. **, $P < 0.01$; ****, $P < 0.0001$; determined by two-sample t-test in Origin. Source data are provided as a Source Data file.

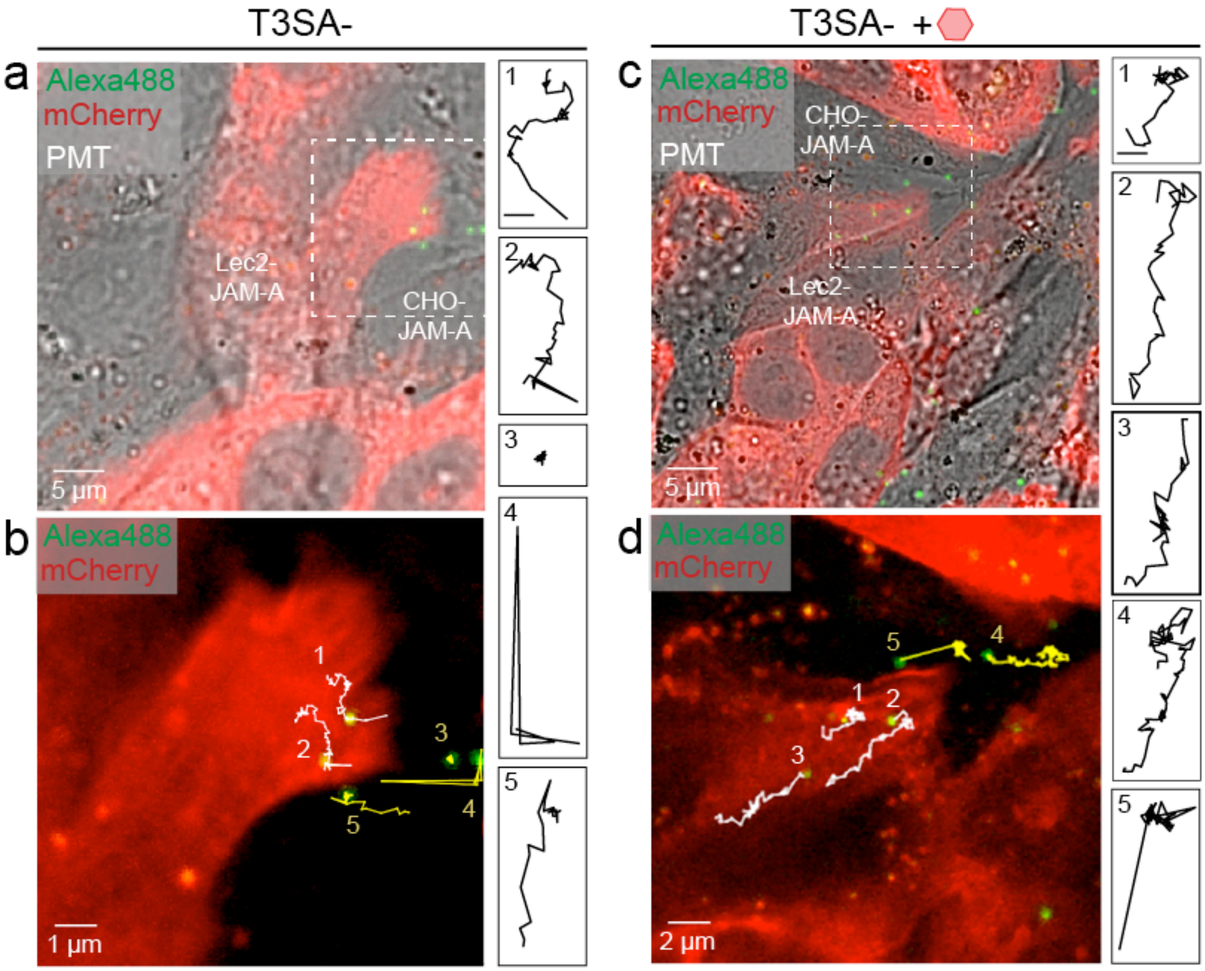

Supplementary Figure 9| Real-time confocal fluorescence imaging of Alexa 488-labeled T3SA- reovirus incubated on co-culture of CHO-JAM-A and Lec2-JAM-A cells in the absence (a, b) and presence (c, d) of 1 mM Neu5Ac. (**a, c**) Overlay images of Alexa 488 (virions), mCherry (actin of Lec2-Jam-A), and PMT signals. (**b, d**) Time-lapse trajectories of T3SA- particles. White and yellow trajectories represent virion movement on Lec2-JAM-A cells and CHO-JAM-A cells, respectively. A magnification of each trajectory is shown on the right side with the corresponding number (scale bar: 1 µm). T3SA- particles diffuse on both cell types to a similar extent and independent of the addition of 1 mM Neu5Ac, due to the lack of SA binding by T3SA-.

References:

1. Dietrich, M.H.; Ogden, K.M.; Long, J.M.; Ebenhoch, R.; Thor, A.; Dermody, T.S.; Stehle, T. Structural and functional features of the reovirus σ1 tail. *J. Virol.* **2018**, JVI. 00336-00318.
