# Supplementary material for: Glycan-mediated enhancement of reovirus receptor binding
Source: Nat Commun. 2019 Oct 1;10:4460. doi: 10.1038/s41467-019-12411-2 (PMC6773860; doi:10.1038/s41467-019-12411-2)
Supplement: Supplementary file 3 — Description of Additional Supplementary Files [file 41467_2019_12411_MOESM3_ESM.docx]

**Description of Additional Supplementary Files**

**File Name: Supplementary Movie 1**

**Description:** Single-virus tracking of T3SA+ binding to cocultured CHO-JAM-A and Lec2-JAM-A cells in the absence of Neu5Ac. Virus traces on CHO-JAM-A cells (unlabeled) are colored in black and on Lec2-JAM-A cells (labeled with mCherry) in white. Snapshots of this video are shown in Figure 6b and c. During real-time imaging, Alexa 488-labeled viral particles were recorded with a frame time of 13.32 s for a total time of ~ 30 min. The movie speed is ~ 90x real-time.

**File Name: Supplementary Movie 2**

**Description:** Single-virus tracking of T3SA+ binding to cocultured CHO-JAM-A and Lec2-JAM-A cells in the presence of Neu5Ac. Virus traces on CHO-JAM-A cells (unlabeled) are colored in black and on Lec2-JAM-A cells (labeled with mCherry) in white. Snapshots of this video are shown in Figure 6d and e. During real-time imaging, Alexa 488-labeled viral particles were recorded with a frame time of 13.32 s for a total time of ~ 30 min. The movie speed is ~ 110x real-time.

**File Name: Supplementary Movie 3**

**Description:** Single-virus tracking of T3SA- binding to cocultured CHO-JAM-A and Lec2-JAM-A cells in the absence of Neu5Ac. Virus traces on CHO-JAM-A cells (unlabeled) are colored in black and on Lec2-JAM-A cells (labeled with mCherry) in white. Snapshots of this video are shown in Supplementary Figure 9. During real-time imaging, Alexa 488-labeled viral particles were recorded with a frame time of 13.32 s for a total time of ~ 30 min. The movie speed is ~ 110x real-time.

**File Name: Supplementary Movie 4**

**Description:** Single-virus tracking of T3SA- binding to cocultured CHO-JAM-A and Lec2-JAM-A cells in the presence of Neu5Ac. Virus traces on CHO-JAM-A cells (unlabeled) are colored in black and on Lec2-JAM-A cells (labeled with mCherry) in white. Snapshots of this video are shown in Supplementary Figure 9. During real-time imaging, Alexa 488-labeled viral particles were recorded with a frame time of 13.32 s for a total time of ~ 30 min. The movie speed is ~ 110x real-time.
